# Opposing Regulation of Cancer Properties via KRT19-Mediated Differential Modulation of Wnt/β-Catenin/Notch Signaling in Breast and Colon Cancers

**DOI:** 10.3390/cancers11010099

**Published:** 2019-01-15

**Authors:** Subbroto Kumar Saha, Yingfu Yin, Hee Sung Chae, Ssang-Goo Cho

**Affiliations:** Department of Stem Cell & Regenerative Biotechnology, Incurable Disease Animal Model & Stem Cell Institute (IDASI), Konkuk University, Seoul 05029, Korea; subbroto@konkuk.ac.kr (S.K.S.); yfy_21@hotmail.com (Y.Y.); gmltjdgk@naver.com (H.S.C.)

**Keywords:** KRT19, Wnt/β-catenin, NUMB crosstalk, Notch pathway, colon cancer, breast cancer

## Abstract

Although Keratin 19 (KRT19) has been reported as a tumor cell marker and found to interact with other proteins that modulate cancer properties, its role in cancer prognosis remains to be fully elucidated. We found that *KRT19* expression was increased in both colon and breast cancer, but that knockdown of *KRT19* showed opposing effects on cancer properties. In colon cancer, *KRT19* knockdown resulted in suppression of cancer via downregulation of Wnt/Notch signaling without altering *NUMB* transcription. In breast cancer, *KRT19* knockdown led to an increase in cancer properties because of attenuated Wnt and enhanced Notch signaling. In colon cancer, KRT19 interacted with β-catenin but not with RAC1, allowing the LEF/TCF transcription factor to bind primarily to the *LEF1* and *TCF7* promoter regions, whereas in breast cancer, KRT19 interacted with the β-catenin/RAC1 complex and led to apparent upregulation of *NUMB* expression and NUMB-mediated suppression of Notch signaling. These results reveal a novel differential role of KRT19 in carcinogenesis, due to differential modulation of Wnt/β-catenin/Notch signaling crosstalk through various interactions of KRT19 with only β-catenin or with the β-catenin/RAC1 complex, which might have implications for clinical cancer research.

## 1. Introduction

Breast cancer (BC) and colorectal cancer (CRC) are commonly diagnosed with substantially high incidence rates worldwide, including Republic of Korea [1,2,3,4]. Over the past few decades, cancer treatment has become an important area for molecular research, with contributions to our understanding of vital mechanisms underlying key phenomena in cancer progression [5]. Substantial effort is still needed to identify novel biomarkers and/or drugs that can cure these cancers.

Keratins (KRTs) have been reported as widely detectable tumor markers [6]. Some KRT members, for example KRT8 and KRT18, are preferably expressed in cancer cells and serve as critical markers of epithelial-mesenchymal transition (EMT) via the PI3K/AKT/NF-*k*B axis [7]. Additionally, KRT17 has also been identified as an oncoprotein in cervical cancer [8]. Based on their interesting roles in cancer progression, there is an increasing focus on the molecular basis of the roles of keratins in cancer.

KRT19 (the smallest type I KRT protein, ~40 kDa) is another member of the keratin family [9], which shows differential expression patterns in a range of cell types in the epithelium [10,11,12]. Recent studies indicate that KRT19 can regulate cancer properties by modulating signaling pathway cascades such as the EGR1/PTEN/AKT, Wnt/β-catenin/Notch, and other pathways [13,14], with knockdown of *KRT19* promoting breast cancer cell proliferation, migration, and sphere formation through NUMB-dependent crosstalk in the Wnt/Notch signaling pathway [13] and activation of AKT signaling [14]. Moreover, KRT19 transcription was reported to be augmented by activation of the HER2/ERK/SP1 signaling pathway, resulting in translocation of KRT19 to the HER2 receptor; consequently, HER2 activation was stabilized in breast and lung cancers [15,16]. Alternatively, silencing of *KRT19* inhibited hepatocellular cancer (HCC) and cancer stem cell progression by correlating with oncogenic microRNAs, invasive/metastasis markers, and the TGFβ/Smad signaling pathway [17,18,19]. Moreover, progression of HCC can be modulated through the PDGFRα-laminin B1-KRT19 signaling cascade, and this can promote early recurrence, metastasis, and microvascular invasion in HCC [17,20,21]. Moreover, KRT19^+^ colon cancer stem cells showed high radio-resistance by raising LGR5^+^ crypt-based columnar cells in the colon and intestines [22]. Recently, we showed that KRT19 has the ability to reprogram breast cancer [23]. Thus, the molecular mechanism underlying the contradictory roles of KRT19 in various cancers needs to be examined to reveal the function of KRT19 in specific cancers.

Here, we found that silencing of *KRT19* led to decreased cell proliferation, migration, and sphere formation in colon cancer and that these phenomena contrasted with those found in breast cancer. We suggest the differential molecular mechanisms that might explain the contradictory roles of KRT19 in breast and colon cancer cells, which might contribute to the design of new cancer therapies.

## 2. Results

### 2.1. KRTs Are Differentially Expressed in Colon and Breast Cancers

**Figure 1:**
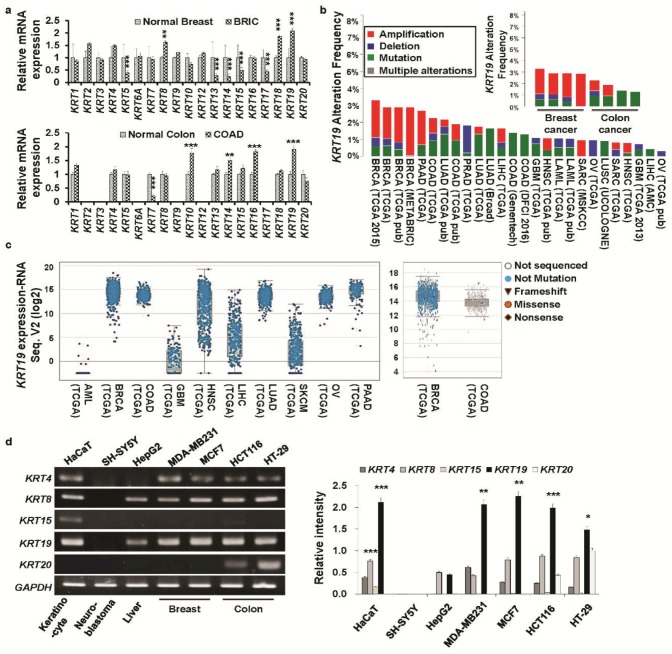
Expression of different Keratins (*KRTs*) family genes in various cancers. (**a**) Relative expression changes in *KRT* family genes in colon (COAD—colon adenocarcinoma) and breast (BRIC—breast invasive carcinoma) cancer compared to their normal counterparts; the data were extracted from two independent studies (Gluck breast, *n* = 158; Ki colon, *n* = 78) from the Oncomine database (Gluck et al. [24], Ki et al. [25]). All data are presented as the mean ± SEM. (** *p* < 0.01, *** *p* < 0.001). (**b**) Percent (%) alteration frequency of *KRT19* in several cancer types. Data were acquired from The Cancer Genome Atlas (TCGA) portal: http://www.cbioportal.org. (**c**) Analysis of RNA sequencing data of *KRT19* expression levels in 10 types of human cancers from the cBioPortal database (http://www.cbioportal.org). Every spot represents a single study, with white spots representing those analyzed without gene sequencing and blue spots representing normal results of gene sequencing. The levels of *KRT19* expression in breast and colorectal cancer (right panel). The median and interquartile ranges are presented. (**d**) The expression of several keratin family members was analyzed by RT-PCR in different cancer cell lines. *GAPDH* was used as an internal standard, and the bar diagram shows relative expression levels (right panel). Each experiment was performed in triplicate, and data are represented as means ± standard deviations (SD) (* *p* < 0.05, ** *p* < 0.01, *** *p* < 0.001). (Abbreviations: BRCA: Breast invasive carcinoma; PAAD-Pancreatic adenocarcinoma; COAD: Colon adenocarcinoma; LUAD: Lung adenocarcinoma; LIHC: Liver hepatocellular carcinoma; LUSC: Lung squamous cell carcinoma; GBM: Glioblastoma multiforme; HNSC: Head and neck squamous cell carcinoma; LAML: Acute myeloid leukemia; SARC: Sarcoma; OV: Ovarian serous cystadenocarcinoma).

In our previous study, we found that KRT19 was obligatory for cancer and cancer stem cell progression because of its selective regulation of the NUMB-dependent Notch signaling pathway [13]. KRT19 is also a differential regulatory factor implicated in cancer progression [14,17,18,19]. Its role regarding cancer prognosis remains to be fully elucidated. Therefore, from the Oncomine database (www.oncomine.com), we determined that *KRT* family genes (*KRT 1–20*) were differentially expressed in both colon or breast cancers compared to respective normal tissues (Figure 1a, Figures S1 and S2). Among the *KRT*s, *KRT19* was significantly (*p* < 0.001) overexpressed in both colon (COAD) and breast (BRIC) cancers compared to that in normal tissues (Figure 1a). Moreover, we scrutinized the percent (%) alteration frequency of *KRT19* in various cancers from TCGA data (http://cancergenome.nih.gov/) using the cBioPortal web (http://www.cbioportal.org/). We found that *KRT19* displayed high alteration frequency in numerous cancer patients and that *KRT19* was frequently amplified in up to 3.5% of breast and colon cancer cases (Figure 1b). Additionally, RNA sequencing data from the TCGA database showed upregulation of *KRT19* mRNA expression in several cancer types. Specially, *KRT19* mRNA expression was upregulated in breast and colorectal cancers (Figure 1c).

### 2.2. Knockdown of KRT19 Differentially Regulates Properties of Colon and Breast Cancers

We then analyzed expression patterns of *KRT* family genes in breast cancer cell lines (MCF7 and MDA-MB231) and colon cancer cell lines (HCT116 and HT29), along with immortalized human keratinocytes (HaCaT), neuroblastoma (SH-SY5Y), and hepatocellular carcinoma (HepG2) cells (Figure 1d). We found strong expression of *KRT19* specifically in colon and breast cancer cells.

**Figure 2:**
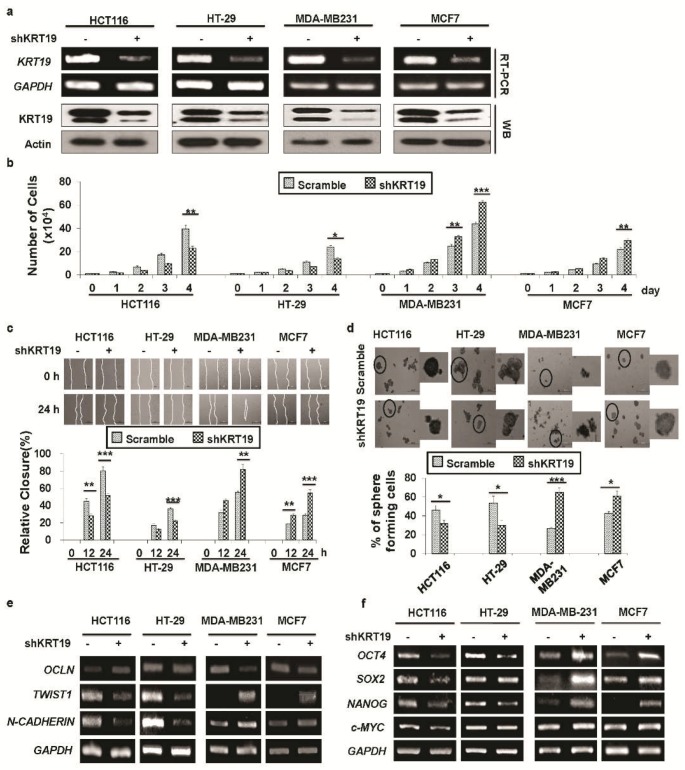
Effect of *KRT19* knockdown on colon and breast cancer proliferation, migration, and sphere formation. (**a**) *KRT19* expression in scramble and shKRT19 cells was analyzed by RT-PCR and Western blotting in the indicated cancer cell lines. *GAPDH*/actin was used as a loading control. (**b**) Proliferation of the indicated cells analyzed by trypan blue cell counting. Cells were enumerated up to 4 days. (**c**) Wound healing migration assay used to assess the migration capacity of the indicated cells. Cell migration was observed at the indicated time point and is shown as percent (%) enclosure. Photos were taken under an inverted light microscope. Scale Bar: 500 μm. (**d**) Cell sphere formation assay performed in the non-coated culture dish. Spheres were enumerated after 5 days of culture with crystal violet then photos were taken under an inverted light microscope. The black circular areas in the left panel photos were enlarged in the right panel, and data are presented as percent (%) of sphere forming cells. Scale Bar: 500 μm. (**e**) RT-PCR analysis of the epithelial-mesenchymal transition (EMT) and mesenchymal-epithelial transition (MET) markers. (**f**) mRNA expression of stemness markers analyzed by RT-PCR. Each experiment was repeated at least three times, and data are represented as means ± SDs (* *p* < 0.05, ** *p* < 0.01, *** *p* < 0.001).

Then, we tried to silence *KRT19* expression by transducing control shRNA (scramble) and *KRT19* targeted shRNA (shKRT19) into both colon and breast cancer cell lines (i.e., HCT116, HT29, MDA-MB231, and MCF7 cells), and the knockdown effect was confirmed by RT-PCR and Western blot analyses (Figure 2a). Although knockdown of *KRT19* increased cancer properties in breast cancer cells, suppression of *KRT19* expression showed the opposite effect in colon cancer cells (Figure 2b–f). In detail, silencing of *KRT19* expression led to decreased cell proliferation (Figure 2b), migration (Figure 2c), and sphere formation (Figure 2d) in colon cancer cells, although the opposite effect was observed in breast cancer cells upon *KRT19* knockdown (Figure 2b–d). We also checked EMT and stemness marker expression and found that, upon *KRT19* knockdown, expression of EMT and stemness marker genes was downregulated in colon cancers, whereas the expression of markers was upregulated in breast cancers upon knockdown of *KRT19*, relative to their respective control (scramble) cells (Figure 2e,f), suggesting differential roles of *KRT19* in various cancer cells.

**Figure 3:**
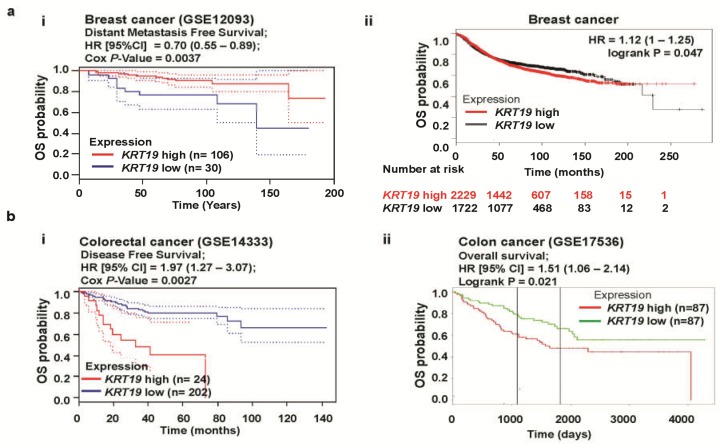
Correlation of *KRT19* gene expression with clinical outcomes in breast and colon cancers. (**a**) The survival curve comparing patients with high (red) and low (blue/black) expression in breast cancer was plotted from the PrognoScan (**ai**) and Kaplan Meier plotter (**aii**) databases. Dotted lines indicate the 95% confidence intervals for each group. Survival curve analysis was conducted using a threshold Cox *p*-value < 0.05. (**b**) Survival curve comparing a patient with high (red) and low (blue/green) expression of *KRT19* in colon cancers plotted with data from the PrognoScan (**bi**) and PROGgeneV2 (**bii**) databases. Dotted lines indicate the 95% confidence intervals for each group. Survival curve analysis was conducted using a threshold Cox *p*-value < 0.05.

Next, to investigate the clinical significance of *KRT19* in different cancer patients, we conducted a computational analysis of overall survival (OS) probability, which has been associated with *KRT19* expression in cancer patients based on various web programs, including PrognoScan (http://dna00.bio.kyutech.ac.jp/PrognoScan/) [26], Kaplan-Meier Plotter (KMplot) (www.kmplot.com) [27], and PROGgeneV2-Pan Cancer Prognostics Database (http://www.compbio.iupui.edu/proggene) [28]. We revealed that high expression of *KRT19* (median values as the cutoff) was significantly correlated with good outcomes in breast cancer, whereas a poor prognosis was associated with colon cancer patients with high *KRT19* expression (Figure 3a,b). Moreover, KMplot confirmed different clinical outcomes regarding *KRT19* expression in various cancers, including a good prognosis with gastric cancer (*n* = 876, hazard ratio (HR) = 0.83, 95% confidence interval (CI) = 0.7–0.98, *p* = 0.026), whereas lung (*n* = 1926, hazard ratio (HR) = 1.31, 95% confidence interval (CI) = 1.15–1.48, *p* = 3.6e-05) and ovarian (*n* = 1306, hazard ratio (HR) = 1.09, 95% confidence interval (CI) = 0.96–1.24, *p* = 0.19) cancers were associated with poor outcomes (Figure S3). Therefore, these data strongly indicate that *KRT19* expression might play conflicting roles in cancer prognosis.

### 2.3. KRT19 Differentially Modulates Wnt/β-Catenin/Notch Signaling Pathways in Colon and Breast Cancers

**Figure 4:**
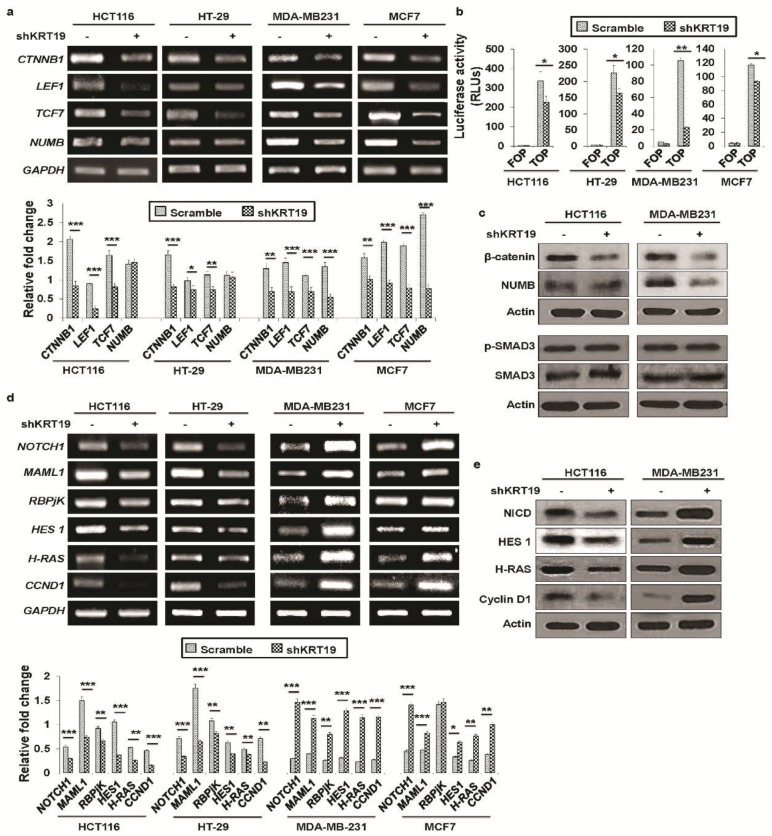
*KRT19* differentially regulates Wnt/β-catenin/Notch signaling in colon and breast cancers. (**a**) Expression of Wnt signaling-related genes in the indicated cells was quantified by RT-PCR. Bands were quantified by scanning densitometry analysis and normalized to that of *GAPDH* (lower panel). (**b**) Wnt signaling transcriptional activity was measured using a FOP- and TOP-Flash reporter assay and is shown in relative luciferase units (RLUs). (**c**) Expression of β-catenin, NUMB, p-SMAD3, and SMAD3 proteins in the indicated cells was analyzed by Western blotting. Actin was used as an internal standard. (**d**) Relative expression levels of Notch signaling-related genes in colon and breast cancer was determined by RT-PCR. Bands were quantified by scanning densitometry analysis and normalized to that of *GAPDH* (lower panel). (**e**) Expression of NICD, HES1, H-RAS, and Cyclin D1 proteins in the indicated cells was analyzed by Western blotting. Actin was used as an internal standard. Error bars represent ± SDs of the means of three independent experiments (* *p* < 0.05, ** *p* < 0.01, *** *p* < 0.001).

In our previous study, we showed crosstalk between Wnt/β-catenin and *Notch* signaling in breast cancer progression [13]. As we found contrasting effects of *KRT19* knockdown in breast and colon cancer cells, we compared the signaling mechanisms involved in this study. At first, we examined the expression of Wnt/β-catenin signaling-related genes and found that expression levels of *CTNNB1*, *LEF1*, and *TCF* genes were significantly downregulated both in colon and breast cancers (Figure 4a), which was confirmed by a TOP-/FOP-Flash reporter assay (Figure 4b). The expression of β-catenin protein was also attenuated both in colon and breast cancers in *KRT19* knockdown cells (Figure 4c). It is reported that SMAD3 could be an interacting partner of β-catenin for processing canonical Wnt signaling [29,30,31]. Thus, we checked the expression of p-SMAD3 in *KRT19* knockdown colon and breast cancer cells, but there was no significant change observed upon *KRT19* knockdown (Figure 4c), suggesting that β-catenin/SMAD3 mediated Wnt signaling activation was not involved in this phenomenon. We then checked the expression of NUMB, another Wnt/β-catenin signaling-related protein, which did not show any apparent change in transcription as well as translation level in colon cancer cells upon *KRT19* knockdown, whereas significantly decreased expression was found in breast cancer cells upon *KRT19* knockdown (Figure 4a,c). As NUMB works an upstream inhibitor of the Notch signaling pathway [32,33,34,35,36], we also examined expression of Notch signaling-related genes in colon and breast cancer cells. Interestingly, we revealed that although expression of several Notch signaling pathway-related genes such as *NOTCH1*, *MAML1*, *RBPjK*, *HES1*, *H-RAS*, and *CCND1* was significantly increased upon *KRT19* knockdown in breast cancer, colon cancer cells showed the opposite effect in the expression of Notch signaling pathway-related genes upon *KRT19* knockdown (Figure 4d). The opposite effect in the expression of Notch signaling pathway-related proteins was also confirmed by Western blot in *KRT19* knockdown cells (Figure 4e), suggesting that NUMB might play an important role in the biased effect of KRT19 in Wnt/Notch signaling crosstalk in colon and breast cancer cells.

### 2.4. KRT19 Regulates β-Catenin Localization and Differential RAC1 Localization

The evidence described above strongly suggested that KRT19 might be dispensable for β-catenin translocation. In the canonical Wnt pathway, when the Wnt protein is recruited to the Frizzled receptor, which is also termed Wnt-on, transcription of Wnt targets is stimulated [37]. Since β-catenin has been implicated in many routes to regulation of transcriptional activity, among which its nuclear import, which has become a well-characterized model [38]. In this study, we found that the transcription level of β-catenin was suppressed dramatically upon *KRT19* knockdown in colon and breast cancer cells (see Figure 4a). Hence, we supposed that the cytoplasmic and nuclear distribution pattern of β-catenin is dysregulated upon *KRT19* suppression.

**Figure 5:**
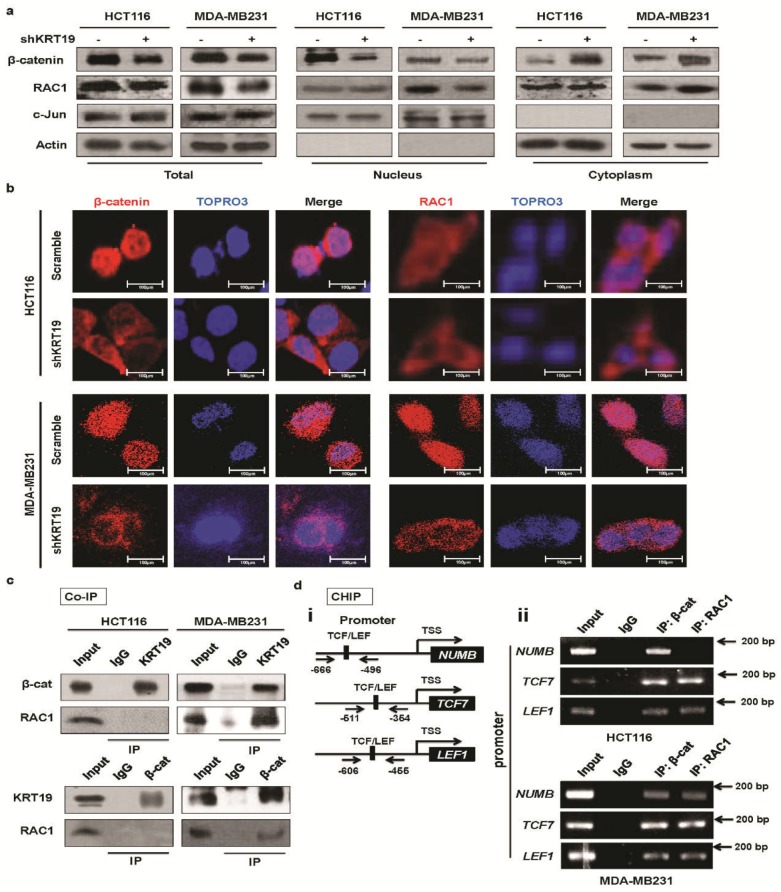
KRT19 differentially regulates β-catenin/RAC1 localization and directly interacts with either β-catenin or the β-catenin/RAC1 complex in colon and breast cancer cell lines. (**a**) Cell fractionation assay for β-catenin and RAC1 analyzed by Western blot; actin and c-Jun were used as the cytoplasmic and nuclear markers, respectively, in the indicated cells. (**b**) β-catenin and RAC1 subcellular localizations were analyzed using an immunocytochemistry (ICC) assay in the indicated cells. Photos were taken under an inverted confocal microscope. Scale bar used in photos is 100 µm. Red: β-catenin/RAC1; Purple: TOPRO3. (**c**) Co-immunoprecipitation (Co-IP) was performed using Protein A/G Sepharose and antibodies specific for KRT19, β-catenin, and RAC1, or normal IgG in the indicated cancer cells. Cell lysates were analyzed by Western blot. (**d**) Schematic representation of *NUMB*, *TCF7*, and *LEF1* promoters that contain TCF/LEF binding sequences (**i**). Expression of *NUMB*, *TCF7*, and *LEF1* promoters was analyzed in the indicated cells by RT-PCR using a ChIP assay after pulldown of normal IgG, β-catenin, and RAC1 (**ii**). Sonicated chromatin lysate was used as the input.

In breast cancer cells, both nuclear RAC1 and β-catenin levels decreased upon *KRT19* knockdown [13], which supports the evidence that RAC1 directly regulates β-catenin nuclear translocation [39,40]. Nevertheless, in colon cancer cells, despite the similar effect of *KRT19* on β-catenin nuclear translocation, nuclear RAC1 levels did not decrease upon *KRT19* knockdown (Figure 5a). Moreover, although the cytoplasmic and nuclear distribution patterns of both β-catenin and RAC1 were dysregulated upon *KRT19* suppression in breast cancer cells, and *KRT19* knockdown led to a dramatic decrease in nuclear β-catenin and RAC1 levels, in colon cancer cells, *KRT19* knockdown resulted in dysregulation of β-catenin localization but had no effect on RAC1 localization (Figure 5a). In colon cancer cells, the cytoplasmic and nuclear distribution patterns of RAC1 were maintained, regardless of whether *KRT19* was knocked down. Additionally, we checked transcription levels of *RAC1* in breast and colon cancer cells and revealed that the mRNA level of *RAC1* did not change in colon cancer cells, whereas *RAC1* mRNA levels were significantly downregulated in breast cancer cells (Figure S4). Concurrently, RAC1 and β-catenin translocation were confirmed through immunocytochemistry (ICC). From the ICC data, we determined that nuclear distribution of β-catenin was significantly decreased in both colon and breast cancer cells, whereas the localization of RAC1, the interacting partner of β-catenin, did not change in colon cancer cells, but its nuclear distribution decreased in breast cancer cells (Figure 5b). Therefore, it might be stated that KRT19-mediated β-catenin nuclear import is differentially regulated by RAC1 translocation. Previous studies validated that RAC1 could augment or dysregulate canonical Wnt signaling through either stabilization or destabilization of β-catenin import [39,40,41]. Thus, these results imply that the differential role of KRT19 might because of discrepant RAC1 nuclear import in colon and breast cancer cells.

### 2.5. RAC1 Differentially Binds to the KRT19/β-Catenin Complex

Before comparing the detailed molecular mechanisms associated with KRT19 expression in colon and breast cancer cells, we hypothesized that whether KRT19 interacts with the β-catenin/RAC1 complex to regulate Wnt/Notch signaling crosstalk. In the case of Wnt-on signaling, β-catenin complex dynamically assembles with many proteins, and core factors are conserved in this assembly [42]. Previously, we reported that KRT19 binds to the β-catenin/RAC1 complex in breast cancer cells [13], and another research group found that RAC1 plays vital roles in β-catenin localization [39,43]. In this current study, we conducted co-immunoprecipitation (Co-IP) and found that KRT19 interacts with β-catenin alone in colon cancer cells and with the β-catenin/RAC1 complex in breast cancer cells (Figure 5c). That is, we could detect almost no interaction between RAC1 and β-catenin or between RAC1 and KRT19. To examine whether the mRNA sequences of *KRT19*/*RAC1* in colon cancer cells are different from those in breast cancer cells, we prepared *RAC1* and *KRT19* cDNA from colon and breast cancer cells. From the sequencing results, we determined that *RAC1* mRNA sequences were the same in both colon and breast cancer cells (Figure S5), whereas the *KRT19* mRNA sequence showed a silent mutation, T→C (on 471 bp), in breast cancer cells alone (Figure S6). Although there were no changes in the amino acid sequence, it also may bias the role of KRT19 in various cancers. Recently, several studies have indicated that silent mutations can play a crucial role in cancer [44,45]. In our study, this silent mutation in *KRT19* may also play a critical role in diverse cancers, but further detailed study might be needed to explore the possible potential role of silent mutation in *KRT19* in diverse cancers.

Next, we performed a ChIP assay to check the transcription activity of *NUMB* and other targets of Wnt/β-catenin signaling. First, we searched for TCF binding site in the *NUMB*, *TCF7*, and *LEF1* promoters (~2 kb upstream) using the Transcription Element Search System (TESS) (University Pennsylvania). In the search, we recognized several TCF sites that could bind β-catenin/RAC1 [46]. To find the relevance of the *NUMB*, *TCF7*, and *LEF1* promoters to β-catenin/RAC1, we performed a ChIP assay using anti-β-catenin/anti-RAC1 antibody in both colon and breast cancer chromatin samples. Fragmented chromatin immunoprecipitated with anti-β-catenin/anti-RAC1 antibody, and immunoprecipitated DNA containing TCF sites was then amplified using selected primers located upstream of the *NUMB*, *TCF7*, and *LEF1* transcription start codons (Figure 5d(i)). The PCR results indicated that both the β-catenin and RAC1 could bind to the promoter region of *NUMB* in breast cancer cells, whereas RAC1 binding to the *NUMB* promoter region was substantially weaker in colon cancer HCT116 cells; however, very strong interaction between β-catenin and the *NUMB* promoter region were detected in HCT116 cells (Figure 5d(ii)). As β-catenin in low amounts bound to the *NUMB* promoter, and no effect was observed on *NUMB* transcription in colon cancer, other factors beyond RAC1 may be involved. However, β-catenin and RAC1 were observed to interact with the *TCF7* or *LEF1* promoter region in both breast and colon cancer cells. Based on these results, we suggest that *NUMB* transcription might be differentially regulated through different binding capacities of other factors, along with RAC1, to the *NUMB* promoter region in breast or colon cancer cells. These results strongly suggest that the contrasting effects of KRT19 in breast and colon cancers might be partially regulated by the differential interactions of KRT19 with β-catenin or the β-catenin/RAC1 complex, which consequently modulates NUMB-dependent Wnt/Notch signaling crosstalk.

## 3. Discussion

KRT19 serves as a detection marker for metastatic epithelial tumors [47,48,49]. However, from the Oncomine and TCGA databases, we found that *KRT19* was differently expressed in various cancer types; specifically, *KRT19* was significantly upregulated in both breast and colon cancers (see Figure 1a–c) [24,25]. Moreover, several studies have shown the contradictory effects of KRT19 in controlling cancer progression, depending on expression patterns and cell types [14,17,18,19]. Moreover, an altered role for KRT19 was observed in HER2^+^/HER2^−^ breast cancer that relied on the level of *KRT19* expression [14,15]. Concurrently, in our previous study, we showed a consistent role for KRT19 in enhancing cancer properties [13,14]. However, the differential role of KRT19 regarding molecular cancer progression and the mechanisms underlying these differences remain elusive.

As we showed in our previous study, knockdown of *KRT19* in breast cancer promoted cancer properties [13], which contrasts with the function of *KRT19* in other cancers [14,19]. In light of previous studies, we aimed to knock down *KRT19* expression in both (breast and colorectal) cancer cell lines using lentiviral-mediated shRNA and the *RRE/REV* lentivirus expressing system [50]. We examined whether *KRT19* in both colon and breast cancers had similar inhibitory effects on breast cancer progression [13,14] or opposite effects [17,18,19]. Our previous study showed that breast cancer properties were intensely upregulated upon *KRT19* knockdown through the upregulation of Notch signaling, which was mediated by the Wnt/β-catenin/NUMB axis [13]. As KRT19 plays a differential role in cancer progression, here, we aimed to compare the roles of KRT19 in colon and breast cancer cells. In this study, we identify *KRT19* as a suppressor of breast cancer, which is consistent with results of our previous study showing that it is an enhancer of colon cancer growth. To uncover the detailed mechanism behind the contrary phenomena, first, we found that cancer properties were differentially regulated upon *KRT19* knockdown; specifically, silencing of *KRT19* led to increased cell proliferation, migration, and sphere formation in breast cancer, whereas the opposite role for *KRT19* was observed in colon cancer. Previously, several studies have shown the differential roles of *KRT19* in proliferation and invasiveness among different cancers such as breast cancer, hepatocellular carcinoma, and colon cancer [14,15,17,18,19]. Additionally, *KRTs* are used prominently as cancer detection markers in RT-PCR [51,52]. These contrary results persuaded us to examine the mechanism underlying the association between KRT19 and cancer progression.

To uncover the molecular mechanisms that define KRT19 function, first, we confirmed results of our previous study. We found that the effect of *KRT19* knockdown on Wnt/β-catenin signaling target genes was quite similar except for NUMB expression in both colon and breast cancer cells. Moreover, we checked another possible signaling pathway, such as TGFβ/SMAD3 pathway, because SMAD3 is one of the interacting partners of β-catenin [29,30,31], which is not involved in our KRT19/β-catenin axis. Expression of p-SMAD3 was not significantly changed upon *KRT19* knockdown in colon and breast cancer cells. Next, we checked Notch signaling in both cancer cells upon *KRT19* silencing. Surprisingly, we found upregulated Notch signaling targeted gene expression in breast cancer cells, whereas this signaling was downregulated in colon cancer cells upon *KRT19* suppression. We also found that the Notch signaling inhibitory protein NUMB was downregulated in breast cancer upon *KRT19* knockdown while no changes were observed in colon cancer (see Figure 4a,c). NUMB is one of the main mediators of Wnt/Notch signaling crosstalk [32,33,34,35]. Additionally, overexpression of *NUMB* can suppress cancer progression through antagonistic effects on Notch signaling [53]. Thus, *NUMB* transcriptional activity might play a pivotal role in the contrasting effects of *KRT19* knockdown in colon and breast cancers. Sequences of *KRT19* mRNA in colon and breast cancers show one silent mutation in breast cancer only (see Figure S6), which might influence *NUMB* transcriptional activity. These results contribute to our knowledge of how *KRT19* differentially regulates cancer properties. Moreover, regulation of β-catenin phosphorylation at Ser33/37/Thr41 or Thr41/Ser45 or Ser552 or Ser675 [54] may be involved in our KRT19/β-catenin/NUMB/Notch signaling cascade which remains to be further elucidated.

In a previous study, NUMB, an upstream inhibitor of the Notch signaling pathway [55] was found to be a downstream effector of β-catenin, based on the fact that β-catenin can bind to the *NUMB* promoter region [46]. It is reported that active RAC1 (GTP-RAC1) could phosphorylate β-catenin at Ser191 and Ser605 by the help of JNK2, subsequently leads to β-catenin nuclear import [41]. Moreover, KRT19-regulated Notch signaling in a *NUMB* transcription-dependent manner was mediated by β-catenin/RAC1 nuclear import [13], although KRT19 regulates Wnt signaling in both cancers without influencing *NUMB* transcription in a colon cancer model (Figure 4a), suggesting a differential mechanism might be involved in the two cancers. Reports on NUMB suggest its diverse function in the modulation of signaling pathways, most of which have focused on its role as a cross-linker between the Wnt/β-catenin and Notch signaling pathways [33,34]. However, Notch signaling can affect cytokine production in a NUMB-independent manner [56]. Recently, intriguing reports on silent mutations have suggested their important roles in cancer [45]. For instance, synonymous somatic mutant BCL2L12 was associated with higher anti-apoptotic activity than the wild type in melanoma [44]. The mRNA sequence of *KRT19* shows one silent mutation, located in the coiled part of its protein structure, in breast cancer but not in colon cancer (Figure S6). A recent study reported that mutations in coils are less robust than those in α-helices and strands [57], this phenomenon may also involve in *KRT19* mutation which needs to be explored in further detail study. However, it is still debatable how Wnt/β-catenin signaling regulates Notch signaling from upstream *KRT19* knockdown.

Mechanistically, in our previous study, we also found that KRT19 interacted with the β-catenin/RAC1 complex in breast cancer and was subsequently imported into the *NUMB* promoter in the nucleus, consequently regulating cancer properties through the Notch signaling pathway [13]. Compared to our previous findings, we found the opposite effect regarding the β-catenin/RAC1 complex; the fraction, ICC, Co-IP, and ChIP assay results confirmed that the role of KRT19 in different cancers is governed by differential mechanisms (see Figure 5). The current study revealed that the differential role of KRT19 in different cancers is due to interactions with either β-catenin alone or with the β-catenin/RAC1 complex, and consequent stabilization and nuclear import of the molecular complex. RAC1 can facilitate β-catenin nuclear translocation signaling, which induces *NUMB* transcription and Notch signaling crosstalk; however, we did not find that RAC1 is associated with β-catenin in colon cancer, perhaps providing supporting evidence for the NUMB-independent phenomenon in colon cancer (see Figure 5c,d). RAC1-β-catenin complexes can move towards the nucleus with the aid of active/inactive RAC1 [41,43,58]. Moreover, this nuclear translocation of a protein complex composed of RAC1B, β-catenin, and Dishevelled (Dvl) can enhance binding with target promoters [40]. In addition, RAC1 along with AKT can phosphorylate β-catenin at Ser552 and Ser675, resulting in translocation of β-catenin to the nucleus [54]. These studies partially validate our anticipated differential mechanisms and suggest that KRT19 directly interacts with either the β-catenin-RAC1 complex to induce nuclear import of β-catenin/RAC1 and to consequently activate *NUMB* transcription and promote Notch signaling crosstalk in breast cancer or β-catenin only, leading to β-catenin stability and nuclear translocation, regardless of RAC1, then Wnt/β-catenin/Notch signaling pathway-mediated colon cancer progression. Thus, further studies are necessary to uncover the effect of β-catenin phosphorylation and RAC1 activation in differential KRT19/β-catenin/NUMB-mediated regulation of Notch signaling crosstalk and contiguous regulation of cancer properties.

## 4. Materials and Methods

### 4.1. Bioinformatics Analysis

The expected expression levels of *KRT* family genes in breast and colon cancers were retrieved from the Oncomine database (https://www.oncomine.org/resource/login.html). The fold changes in mRNA expression in cancer tissue compared to that in their normal counterparts were acquired using a threshold *p*-value of 1E−4, fold change of 2, and gene ranking in the top 10%.

We conducted an integrative analysis of *KRT19* and clinical characteristics using cBioPortal, an open access resource at http://www.cbioportal.org/, which currently provides access to data from more than 48,668 tumor samples and 172 cancer studies in The Cancer Genome Atlas (TCGA, Bethesda, MD, USA) pipeline. The query interface combined with customized data storage enabled us to interactively explore genetic alterations across samples curated from national and international cancer studies and for specific genes. The primary search parameters included alterations (amplification, deep deletion, and missense mutations), copy number alterations (CNAs) from Genomic Identification of Significant Targets in Cancer (GISTIC), and RNA-seq data with the default setting.

Correlations between the expression of *KRT19* and survival in breast and colon cancers were also investigated using the PrognoScan database (http://dna00.bio.kyutech.ac.jp/PrognoScan/). The significant threshold was adjusted to a Cox *p*-value of < 0.05.

Correlations between the expression of *KRT19* and survival in breast, gastric, lung, and ovarian cancers were analyzed in Kaplan–Meier Plotter (http://kmplot.com/analysis/). Kaplan–Meier Plotter can be used to evaluate the effect of 54,675 genes on patient survival using 10,461 cancer samples (5143 breast, 1816 ovarian, 2437 lung, and 1065 gastric cancers) analyzed on the HGU133 Plus 2.0 array (Thermo Fisher Scientific, Waltham, MA, USA). The log rank *p*-value and hazard ratio with 95% confidence intervals were also calculated.

PROGgeneV2 was used to confirm the relationship between the expression of *KRT19* and prognostic outcomes in colon cancer (http://watson.compbio.-iupui.edu/chirayu/proggene/database/index.php). PROGgeneV2 contains data from 134 cohorts that include 21 cancer types. Only data with significant *p*-values were selected for analysis (*p*-value < 0.05).

### 4.2. Cell Culture 

Human colorectal adenocarcinoma cell line HT-29 (ATCC, Manassas, VA, USA), HCT116 (a kind gift from Professor Sung Gu Han, Konkuk University), human breast cancer cell lines (MCF7 and MDA-MB231), hepatocellular carcinoma cells (HepG2), neuroblastoma cells (SH-SY5Y), and immortalized human keratinocytes (HaCaT) (ATCC, Manassas, VA, USA) were cultured in DMEM (HT29, MCF7, HepG2, SH-SY5Y, and HaCaT cell lines) or RPMI 1640 (HCT116 and MDA-MB231 cell lines) (Sigma-Aldrich, Saint Louis, MO, USA) supplemented with 10% heat-inactivated fetal bovine serum (FBS) (Gibco, Thermo Fisher Scientific Ltd., Waltham, MA, USA), 100 U/mL penicillin and 100 mg/mL streptomycin (Gibco). Cells were retained at 37 °C in a humidified atmosphere of 5% CO_2_. Possible mycoplasma contaminations in all cell lines were tested using a BioMycoX^®^ Mycoplasma PCR Detection Kit (Cat. No. CS-D-25) (Cellsafe, Yeongtong-gu, Suwon, Korea) and were authenticated using short tandem repeat (STR) profiling.

### 4.3. Knockdown of KRT19 Using a Short Hairpin RNA (shRNA) Construct 

For knockdown of *KRT19* expression, sense and antisense oligonucleotides were used in the control (scramble) and to target *KRT19* expression [14]. The oligonucleotides were then annealed and cloned into a pGreenPuro lentiviral vector (System Biosciences, Mountain View, CA, USA) containing *BamH*I and *EcoR*I (Takara Bio inc., Kusatsu, Shiga Prefecture, Japan) restriction enzyme sites following the manufacturer’s instructions. Briefly, two oligonucleotides were annealed at 95 °C for 2 min in a heat block with annealing buffer, and then the heat was reduced to allow the samples to reach room temperature. Annealed double-stranded RNA was ligated into a lentiviral vector using the T4 DNA ligase enzyme (Promega, Madison, WI, USA). The newly constructed plasmid was confirmed for sequencing analysis. The sense sequences for the scramble RNA and shKRT19 are 5′-CCTAAGGTTAAGTCGCCCTCGCTC-3′ (non-specific) and 5′-AACCATGAGGAGGAAATCA-3′ (NM_002276.4; 791–809), respectively.

### 4.4. Lentivirus Production and Transduction 

To generate the lentivirus, the RRE/REV lentivirus expressing system [50] was used in this study. Briefly, 60% to 70% confluent HEK293T cells were cultured in 100-mm dishes on the day of transfection using the calcium phosphate transfection method. The medium was replaced with fresh medium and plasmids (RRE, REV and target), and the calcium phosphate mixture was poured dropwise into the dishes. After 12–16 h, the medium containing the plasmids was removed, and the cells were washed once with PBS. Then, an equal amount of medium was added. After 48 h, the cell supernatant (virus soup) was collected and filtered through a 0.45-µm pore capsule and used for infection.

Virus titer was quantified as previously described [59]. For virus infection, we used ~8.0 × 10^8^ IU/mL viral particles for stable knockdown in cells. All of the experiments started at 72 h post infection [60].

### 4.5. RNA Extraction and RT-PCR

Total RNA was extracted using an Easy-Blue RNA Extraction kit (iNtRON Biotechnology, Seongnam-si, Gyeonggi-do, Korea). Total RNA (2 µg) was reverse transcribed into cDNA using a cDNA synthesis kit (Promega, Madison, WI, USA) according the manufacturer’s instructions. The RT-PCR was assessed using r-Taq plus Master Mix (Elpis Biotech, Daejeon, Korea), and the PCR products were analyzed by ~1.5% agarose gel electrophoresis. The bands were separated in agarose gels containing ethidium bromide (EtBr) and observed under UV light. The pictures were analyzed in Photoshop CS6 (Version 13.0.6 x64, San Jose, CA, USA), and the relative expression fold changes were measured in ImageJ. The primers used in this study are shown in Table 1.

### 4.6. Western Blotting

Total cell lysates were extracted from the indicated cells using lysis buffer. Briefly, the cell lysates were held at 4 °C for 15 min, vortexed every 2–3 min, and then centrifuged at 13,000 rpm for 15 min. Afterwards, the concentrations of the extracted proteins were measured using a Bradford assay kit (BioRad, Hercules, CA, USA). Protein samples were loaded onto either 10% or 12% SDS-PAGE gels and then transferred to nitrocellulose blotting membranes. The membranes were blocked with 5% skimmed milk for 1 h and subsequently incubated with primary antibody at 4 °C overnight. The membranes were then washed three times at 10 min intervals with TBS-T buffer and incubated with secondary antibody at room temperature for 1 h. Next, after washing with TBST buffer for 30 min, the membranes were analyzed using an ECL detection kit (Amersham Bioscience, Piscataway, NJ, USA) as described previously [13]. The antibodies used in this study are presented in Table 2.

### 4.7. Cell Proliferation and Viability Assay

For the cell proliferation analysis, control (scramble-transduced) or *KRT19*-knockdown cells (2 × 10^4^ cells/well) were seeded in 12-well plates. Cells were enumerated from 24 h up to the 4-day period using a trypan blue kit. For the cell viability assay, cells were seeded in 96-well plates. At the indicated time point, EZ-cytox reagent (DoGen, Seoul, Korea) was added at a ratio of 1 to 10 and held in an incubator for ~4 h. Afterwards, the relative absorbance was measured at 450 nm using a fluorescence microplate reader.

### 4.8. Wound Healing Cell Migration Assay 

For the wound healing assay, ~90% confluent cells in 60-mm tissue culture dishes were used. The cells were treated with mitomycin C (MMC, 10 µg/mL) for 3 h, and then the cells were scratched with a 200 µL tip. The indicated areas in the dishes were marked, and photos were taken every 12 h. The pictures were analyzed in ImageJ, and the wound closure percentage (%) was determined.

### 4.9. Sphere Formation Assay

The indicated cells (1 × 10^5^ cells) were seeded in non-coated petri dishes with sphere-forming medium (serum-free DMEM/F12 media with B27 supplement, 20 ng/mL epidermal growth factor (EGF) (Sigma-Aldrich, St. Louis, MO, USA), 10 µg/mL insulin (Sigma-Aldrich), and 1% bovine serum albumin (Sigma-Aldrich). After 5 days, colonies were gently collected in conical tubes (SPL Lifesciences) and stained with crystal violet (Sigma-Aldrich). Finally, the colonies were digested with 0.25% trypsin-EDTA (1×) (Gibco, Thermo Fisher Scientific Ltd., Waltham, MA, USA), subsequently enumerated the disassociated cells from the colonies and shown as percent (%) of sphere forming cells.

### 4.10. Luciferase Reporter Assay

For the luciferase assay, the indicated cells (1 × 10^5^ cells/well) were seeded in 12-well plates and transiently transfected with either 1 µg of TOP- or FOP-FLASH with HyliMax transfection reagent (1:3 ratio) (Dojindo, Kumamoto, Japan) according to manufacturer’s instructions [61]. The cells were harvested after 48 h post transfection, and luciferase activity was measured using a luminometer (Veritas microplate luminometer, Tumor Biosystems, CA, USA). Luciferase activity was normalized by β-galactosidase expression levels.

### 4.11. Subcellular Fractionation Assay

Cells were harvested by scraping in cytoplasmic extraction buffer (10 mM HEPES (pH 7.9; Sigma-Aldrich) 10 mM KCl (Sigma-Aldrich), 0.1 mM EDTA (Sigma-Aldrich), 0.1 mM EGTA (Sigma-Aldrich), 1 mM dithiothreitol (DTT, Invitrogen, Carlsbad, CA, USA), and 0.5 mM PMSF (Sigma-Aldrich)). Cells were suspended and agitated 10 min at 4 °C. After subsequent agitation with 0.5% NP-40 (Sigma-Aldrich) for 10 min at 4 °C and then centrifugation at 13,000 rpm at 4 °C, supernatant from the cytoplasmic fraction was transferred to new tubes. Afterwards, the nuclear fraction was extracted using nuclear extraction buffer (20 mM HEPES (pH 7.9), 400 mM NaCl, 1 mM EDTA, 1 mM EGTA, 1 mM DTT, and 1 mM PMSF) with 10 min agitation at 4 °C and subsequently centrifuged at 13,000 rpm for 10 min at 4 °C. Next, the subcellular fraction proteins were assayed by Western blot. Antibodies used in subcellular fraction assay are shown in Table 2.

### 4.12. Immunocytochemistry

Cells (1 × 10^5^ cells/well) were seeded in 12-well plates prior to the experiment. The next day, the cells were washed with ice-cold PBS and fixed with 4% pre-chilled paraformaldehyde for 10 min at room temperature. The cells were then permeabilized using 0.2% Triton X-100 for 10 min at room temperature and blocked with 10% normal goat serum (NGS) (Vector Lab, Burlingame, CA, USA) in PBS for 1 h. Then, the slides were incubated with primary antibody (Table 2) overnight at 4 °C. The following day, the cells were washed thrice with PBS for 5 min each and then incubated with secondary antibody for 1 h at room temperature, followed by three PBS washes for 5 min each. The slides were then counterstained with TOPRO3 (10 ug/Ml, Invitrogen, Carlsbad, CA, USA) for another 10 min. After the cells were washed with PBS an additional three times, followed by mounting and drying, the sections were examined under a Leica TCS SP5 II laser scanning confocal microscopy (Leica Microsystems, Wetzlar, Germany).

### 4.13. Co-Immunoprecipitation (Co-IP) Assay

To analyze protein interactions, a co-immunoprecipitation assay was conducted with the indicated samples. Briefly, 400 µg of cell lysate was pretreated with 30 µL protein A/G agarose beads (Santa Cruz Biotechnology, Dallas, TX, USA) to remove non-specific immunoglobulin. The supernatant was then collected in new tubes and incubated with 3 to 4 µg primary antibodies and immunoglobulin G mouse/rabbit (Table 2) on an agitator overnight at 4 °C. Subsequently, protein A/G agarose was added. The mixture was incubated for an additional 3 h and then spun down at 3000 rpm for 1 min. The pellets were then washed thrice with ice-cold cell lysis buffer, and, subsequently, the immunoprecipitated proteins were analyzed by Western blotting as described above.

### 4.14. Chromatin Immunoprecipitation (ChIP) Assay

To determine protein-DNA interactions, we performed a ChIP assay using a Zymo-Spin^TM^ ChIP assay kit (Zymo Research Corp, CA, USA) according to the manufacturer’s instructions. Briefly, 5 × 10^6^ cells were suspended in ice-cold PBS and then incubated with 1% formaldehyde for 7 min with agitation to allow cross-linking, followed by stopping of cross-linking using 0.125 M glycine with 5 min of agitation. Cross-linked samples were then centrifuged at 3000 × *g* for 1 min at 4 °C, and, after discarding the supernatant, the pellet was re-suspended in lysis buffer with protease inhibitors. Afterwards, the cell lysates were sonicated on ice for 4 cycles (30 sec “ON” and 30 sec “OFF” at 40% amplitude) using a Bandelin Sonopuls HD 2070 sonicator (Bandelin Electronic GmbH & Co., Berlin Germany). The fragmented chromatin was then precleared with protein A-agarose (Santa Cruz Biotechnology, Dallas, TX, USA) for 2 h at 4 °C and centrifuged at 3000 rpm for 1 min. After that, the precleared protein-DNA complexes (400 µg each) were incubated with anti-β-catenin or anti-RAC1 antibody and normal rabbit IgG (control) (Table 2) overnight at 4 °C, followed by centrifugation at 3000 rpm for 1 min. The PCR amplification (30 cycles) was performed with immunoprecipitated DNA using r-Taq Plus Master Mix (Elpis Biotech, Daejeon, Korea) and analyzed by ~1.5% agarose gel electrophoresis. The bands were separated in agarose gels containing EtBr and observed under UV light. The primer sets used for the ChIP assays were as follows: *NUMB*-forward, 5′-TAACCCTTCCCGGGTAACCA-3′, -reverse, 5′-GTTGAGGGTTGGGCAATTCG-3′; *TCF7*-forward, 5′-AGCAGGGGGAATATCTGGTT-3′, -reverse, 5′-CCACTCGGCATAGCCTTAAA-3′; and *LEF1*-forward, 5′-ACCCTCCTCTGCACTTTGG-3′, -reverse, 5′-CTGCGGTAGCTGGCGACT-3′.

### 4.15. Statistical Analysis

All experiments were performed in triplicate. Data were analyzed using GraphPad InStat version 3 program (Sandiego, CA, USA) and presented as the mean ± standard deviation (SD). Statistical analyses were conducted using a two-tailed Student’s *t*-test and analysis of variance (ANOVA) with Turkey–Kramer adjustment to compare the treatment versus the control. Statistical significance was defined as * *p* < 0.05.

## 5. Conclusions

**Figure 6:**
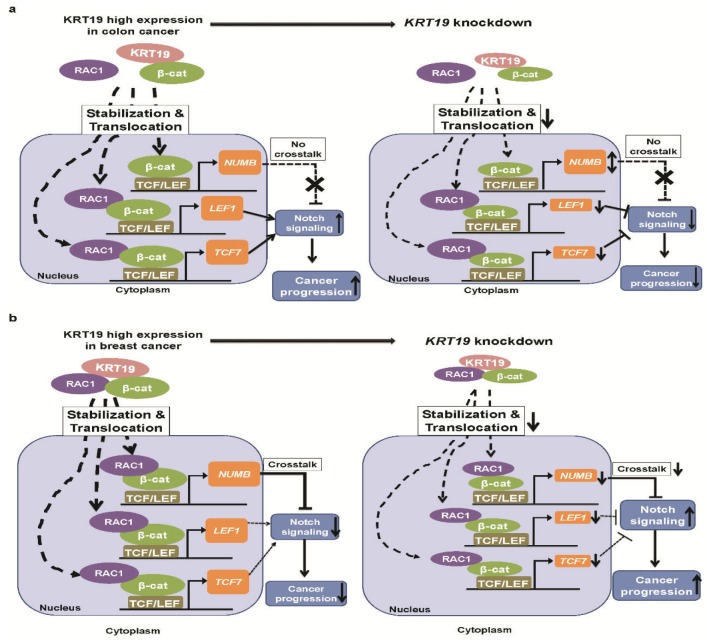
Schematic diagram of the molecular function of *KRT19* as a regulator of cancer properties through differential modulation of the Wnt/β-catenin/Notch signaling pathway in colon and breast cancers. KRT19 directly interacts with only β-catenin to regulate Wnt/Notch signaling pathway and control cancer properties in colorectal cancer (**a**). KRT19 directly binds to the β-catenin/RAC1 complex to regulate *NUMB* transcriptional activity, which is an upstream inhibitor of the Notch signaling pathway and negatively regulates breast cancer properties (**b**).

In conclusion, studies have shown that KRT19 has contradictory effects on tumorigenesis. Thus, we examined the role of *KRT19* in regulating cancer progression as an oncogene or tumor suppressor gene. Through this study, we provide a new perspective on KRT19 in the Wnt/β-catenin/Notch signaling pathway and the differential governance of cancer properties in colon and breast cancers. Moreover, our study suggests that KRT19 directly interacts only with β-catenin and is translocated to *LEF1*/*TCF7* promoter to promote colon cancer progression, whereas KRT19 directly interacts with the β-catenin/RAC1 complex and is predominantly translocated to the *NUMB* promoter in breast cancer and is suppressed through attenuation of Notch signaling (see Figure 6a,b). Therefore, further studies are needed to elucidate the role of KRT19 regarding differences in Wnt/Notch signaling crosstalk in colon and breast cancers. Results of the present study have potential implications for clinical cancer research.

## Figures and Tables

**Table 1:** List of primers used for quantification of specific gene expression.

Accession no.	Gene Name	Forward (5′→3′)	Reverse (5′→3′)
NM_002272.3	*KRT4*	AGATACCTTGGGCAATGACA	CTTGTTCAGGTAGGCAGCAT
*NM_001256282*	*KRT8*	CCTCATCAAGAAGGATGTGGA	CACCACAGATGTGTCCGAGA
*NM_002275*	*KRT15*	AGGGTCAGGAGGAGGATATG	TTTTCTCATTGCCAGAGAGG
NM_002276.4	*KRT19*	TCGAGCATGAGGTATCCAGT	GTAGCGGTTCTTCGTGTCTT
*NM_019010*	*KRT20*	AACGCCAGAACAACGAATAC	CTTCCAGGGTGCTTAACTGA
NM_001005743.1	*NUMB*	TCCCACCTCTCCTACTTCTG	TGCCTCCCCTTCTACTTCTG
NM_001904.3	*CTNNB1*	AAAATGGCAGTGCGTTTAG	TTTGAAGGCAGTCTGTCGTA
NM_003202.3	*TCF7*	GACATCAGCCAGAAGCAAG	CACCAGAACCTAGCATCAAG
NM_016269.4	*LEF1*	CCTGGTCCCCACACAACTG	GGCTCCTGCTCCTTTCTCTG
NM_017617.3	*NOTCH1*	GGGTACAAGTGCGACTGTGA	CGGCAACGTCGTCAATACAC
NM_014757.4	*MAML1*	CACCAGCCACCGAGTAACTT	AACAGGGAGTTCTGCTCGTG
NM_005349.3	*RBPjK*	GAACAAATGGAACGCGATGG	GATGACTTTTATCCGCTTGCTG
NM_005524.3	*HES1*	GGCTAAGGTGTTTGGAGGCT	GGTGGGTTGGGGAGTTTAGG
NM_001130442.1	*H-RAS*	TTCTACACGTTGGTGCGTGA	CACAAGGGAGGCTGCTGAC
NM_053056.2	*CCND1*	CACACGGACTACAGGGGAGT	ATGGTTTCCACTTCGCAGCA
NM_002046.5	*GAPDH*	AATCCCATCACCATCTTCCAG	CACGATACCAAAGTTGTCATGG
EMT markers			
NM_001205255	*OCLN*	CTTCAGGCAGCCTCGTTACA	TCCTCCTCCAGCTCATCACA
NM_000474	*TWIST1*	CTCAGCTACGCCTTCTCG	ACTGTCCATTTTCTCCTTCTCTG
NM_001792	*N-CADHRIN*	GACAATGCCCCTCAAGTGTT	GACAATGCCCCTCAAGTGTT
Stemness markers			
NM_001285986.1	*OCT4*	GTCCCAGGACATCAAAGCTC	CTCCAGGTTGCCTCTCACTC
NM_003106.3	*SOX2*	ACACCAATCCCATCCACACT	GCAAGAAGCCTCTCCTTGAA
NM_024865.3	*NANOG*	ATACCTCAGCCTCCAGCAGA	GCAGGACTGCAGAGATTCCT
NM_002467.4	*c-MYC*	CTCGGATTCTCTGCTCTC	TCGCCTCTTGACATTCTC

**Table 2:** List of antibodies used for quantification of specific protein expression (WB), ICC, Co-IP, and ChIP assays.

Antibody	Catalog No.	Conc. Ratio (WB)	Conc. Ratio (ICC)	Conc. Ratio (Co-IP)	Conc. Ratio (CHIP)
**Primary Ab.**					
Anti-KRT19	SC-53258	1:1000	-	-	-
Anti-Actin	SC-1616	1:1000	-	-	-
Anti-β-catenin	SC-7199	1:2000	1:200	1:50	1:50
Anti-RAC1	SC-217	1:500	1:200	1:50	1:50
Anti-c-Jun	SC-1694	1:1000	-	-	-
Anti-p-SMAD3	9520S	1:1000	-	-	-
Anti-SMAD3	9513S	1:1000	-	-	-
Normal mouse IgG	SC-2025	-	-	1:50	-
Normal rabbit IgG	SC-2027	-	-	1:50	1:50
**Second Ab.**					
Anti-goat	SC-2020	1:1000	-	-	-
Anti-rabbit	SC-2004	1:1000	-	-	-
Anti-mouse	SC-2005	1:1000	-	-	-
Alexa Fluor^®^ 546 (goat anti-rabbit)	A11010	-	1:1000	-	-

## Supplementary Materials

The following are available online at http://www.mdpi.com/2072-6694/11/1/99/s1, Figure S1: KRT family genes expression analysis in colon cancer, Figure S2: KRT gene expression analysis in invasive breast carcinoma, Figure S3: Kaplan-Meier curves for clinical outcomes of patients, Figure S4: RAC1 mRNA expression in scramble and shKRT19 cells analyzed by RT-PCR in the indicated cancer cell lines, Figure S5: RAC1 cDNA sequencing performed using total RNA derived from MDA-MB231 and HCT116 cells, Figure S6: KRT19 cDNA sequencing performed using total RNA derived from MDA-MB231, MCF7, HCT116, and HT29 cells.
