# Supplementary material for: Opposing Regulation of Cancer Properties via KRT19-Mediated Differential Modulation of Wnt/β-Catenin/Notch Signaling in Breast and Colon Cancers
Source: Cancers (Basel). 2019 Jan 15;11(1):99. doi: 10.3390/cancers11010099 (PMC6357186; doi:10.3390/cancers11010099)
Supplement: Supplementary file 1 [file cancers-11-00099-s001.pdf]

cancers

MDPI

Supplementary Materials: Opposing Regulation of Cancer Properties via KRT19-Mediated Differential Modulation of Wnt/β-Catenin/Notch Signaling in Breast and Colon Cancers

Subbroto Kumar Saha, Yingfu Yin, Hee Sung Chae and Ssang-Goo Cho*

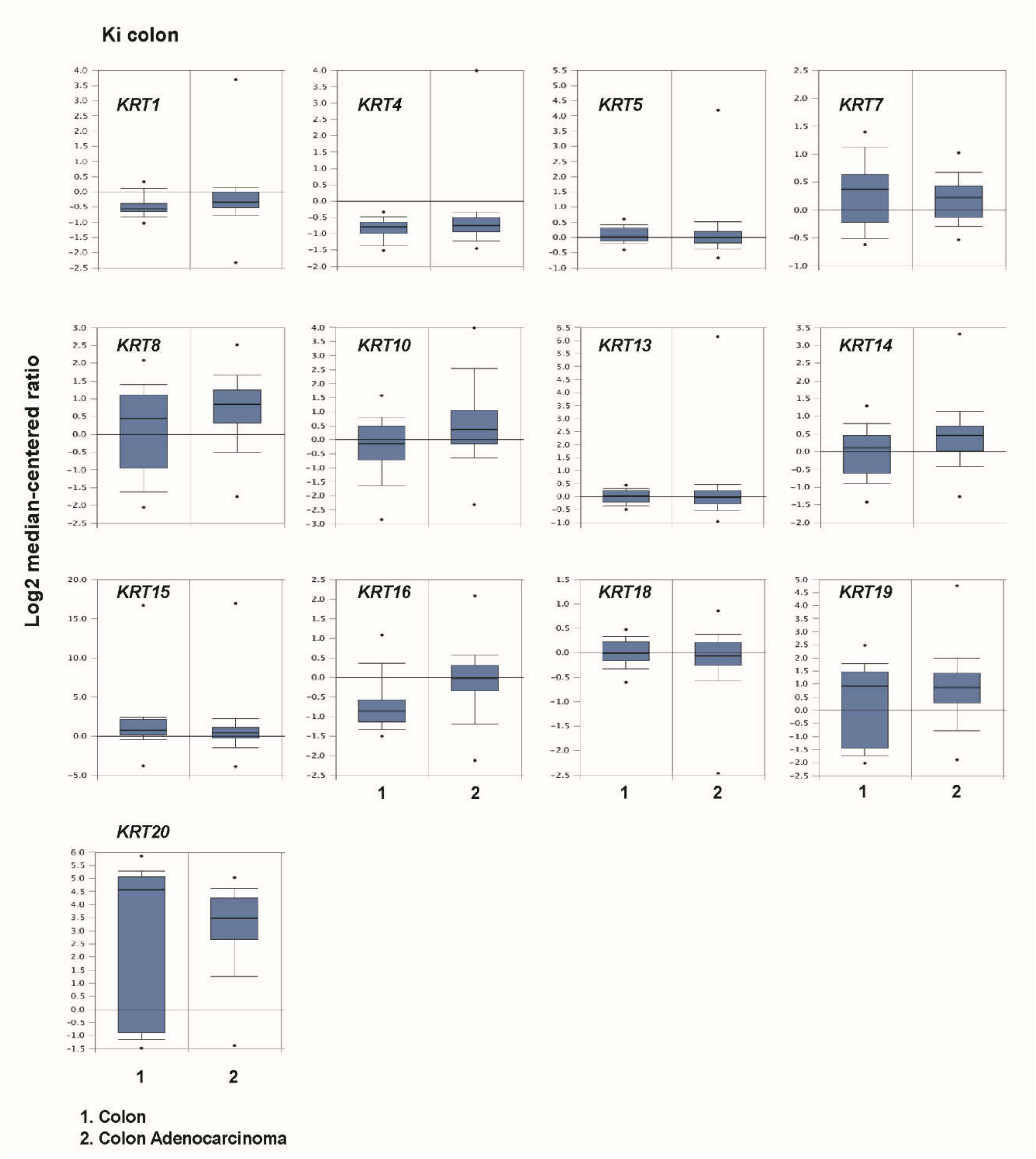

Figure S1. *KRTs* family genes expression analysis in colon cancer. Box plot comparing the expression of specific *KRTs* in normal (left plot) and cancer tissue (right plot) using data from the Oncomine database. Fold change in *KRTs* expression in colon cancer, shown as colon adenocarcinoma relative to normal colon tissue.

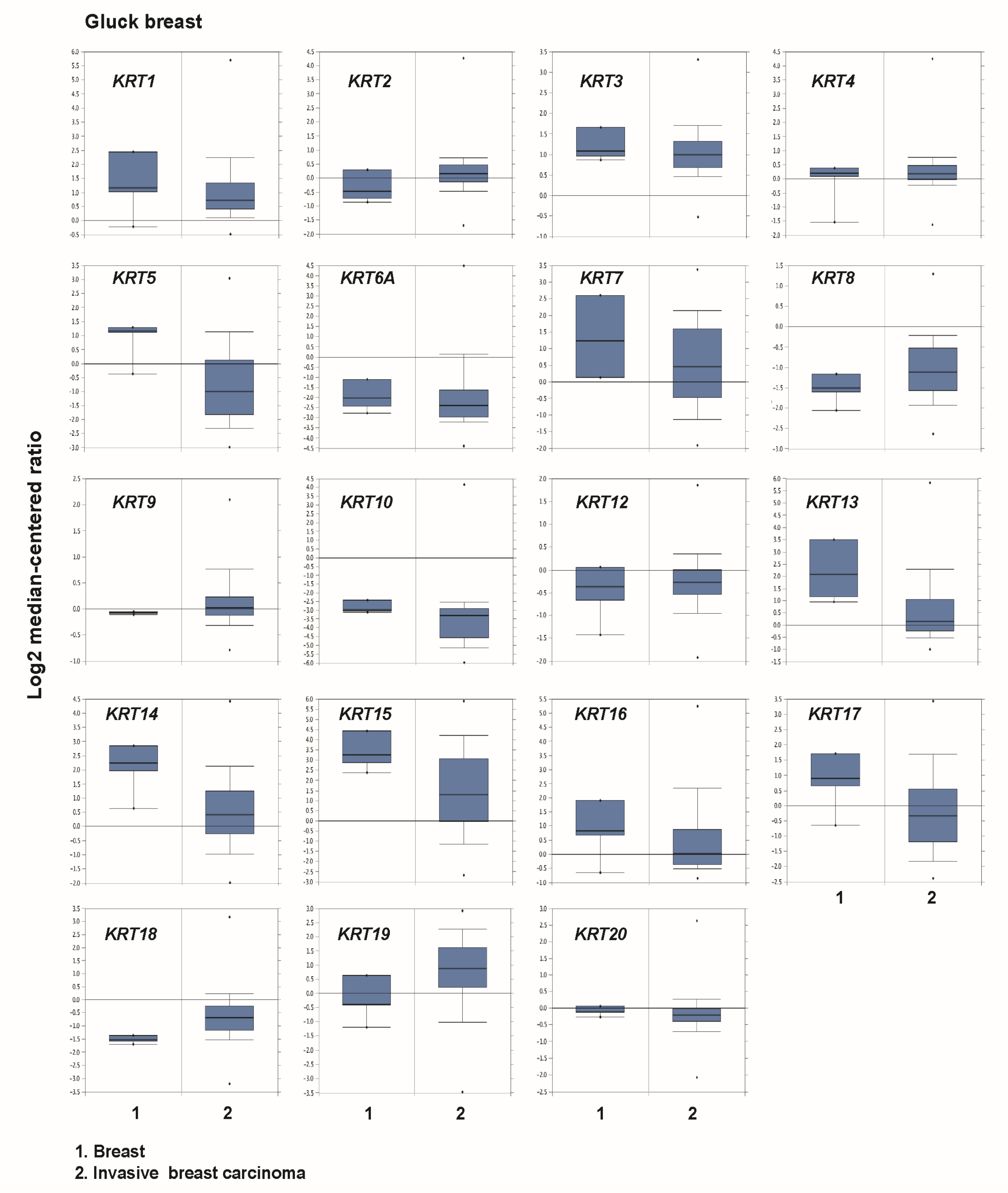

Figure S2. *KRTs* gene expression analysis in invasive breast carcinoma. Box plot comparing the expression of specific *KRTs* in normal (left plot) and cancer tissue (right plot) using data from the Oncomine database. Fold changes in *KRTs* expression in breast cancer, shown as invasive breast carcinoma relative to normal breast.

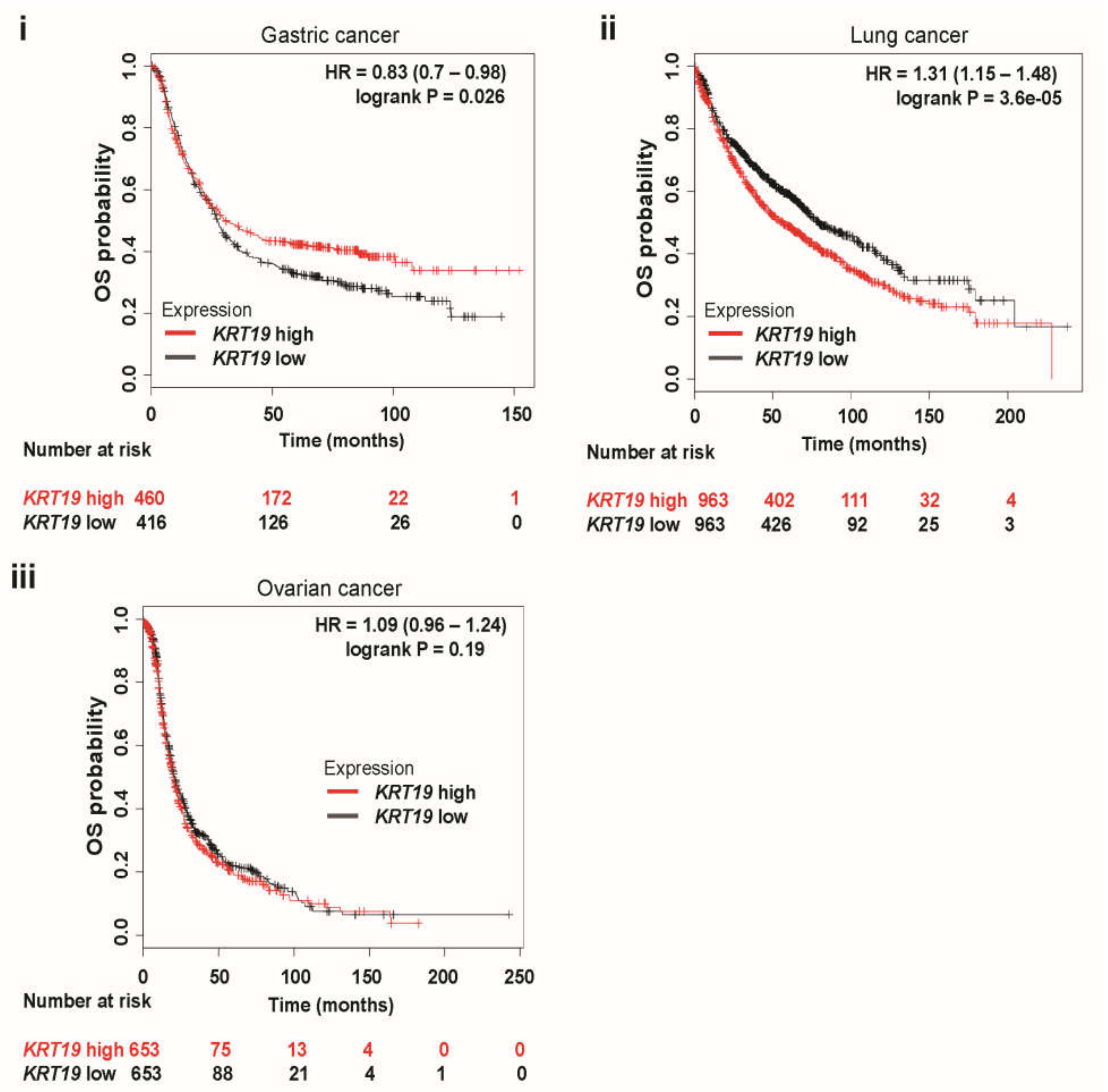

Figure S3. Kaplan-Meier curves for clinical outcomes of patients with (**i**) gastric cancer (n = 876), (**ii**) lung cancer (n = 1926), and (**iii**) ovarian cancer (n = 1306), using Kaplan-Meier Plotter database, with high (red) and low (black) expression levels of *KRT19*.

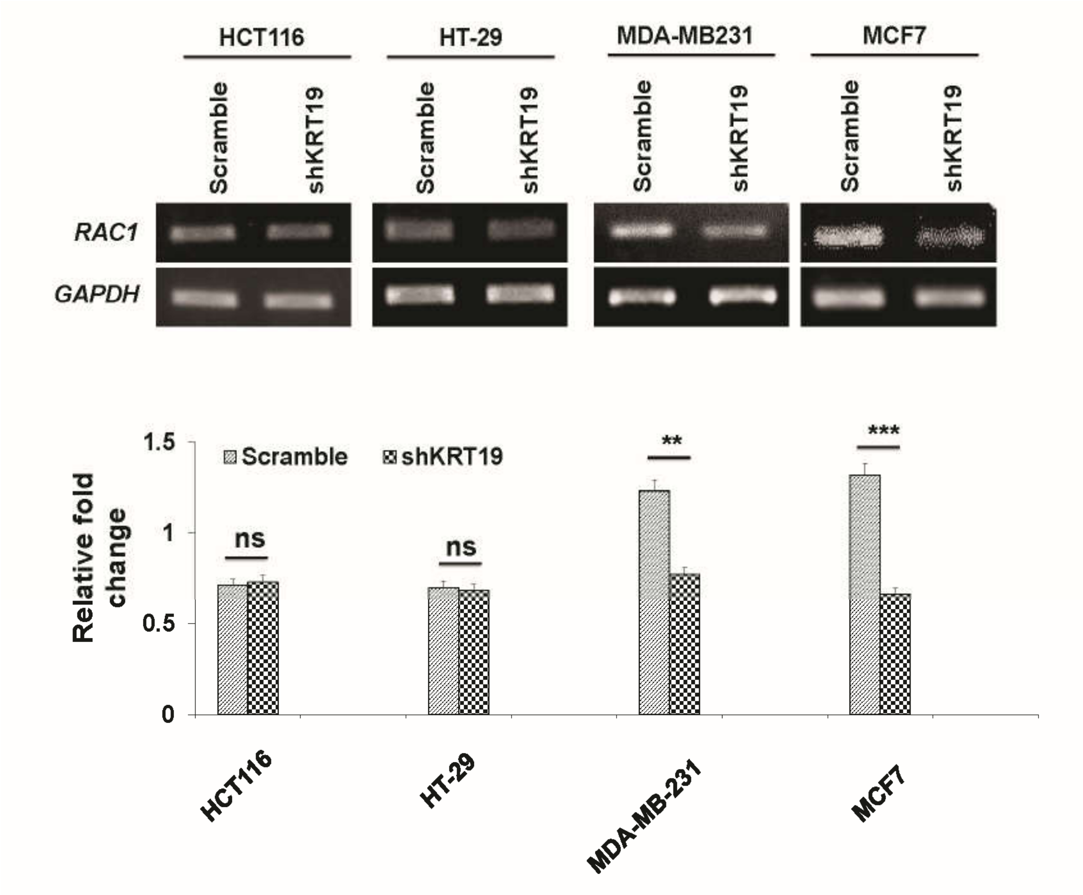

Figure S4. *RAC1* mRNA expression in scramble and shKRT19 cells analyzed by RT-PCR in the indicated cancer cell lines. *GAPDH* was used as a loading control. Bands were quantified by scanning densitometry and normalized to that of *GAPDH* (lower panel). Error bars represent ± SDs of the means of three independent experiments (**$p < 0.01$, ***$p < 0.001$, ns = non-significant).

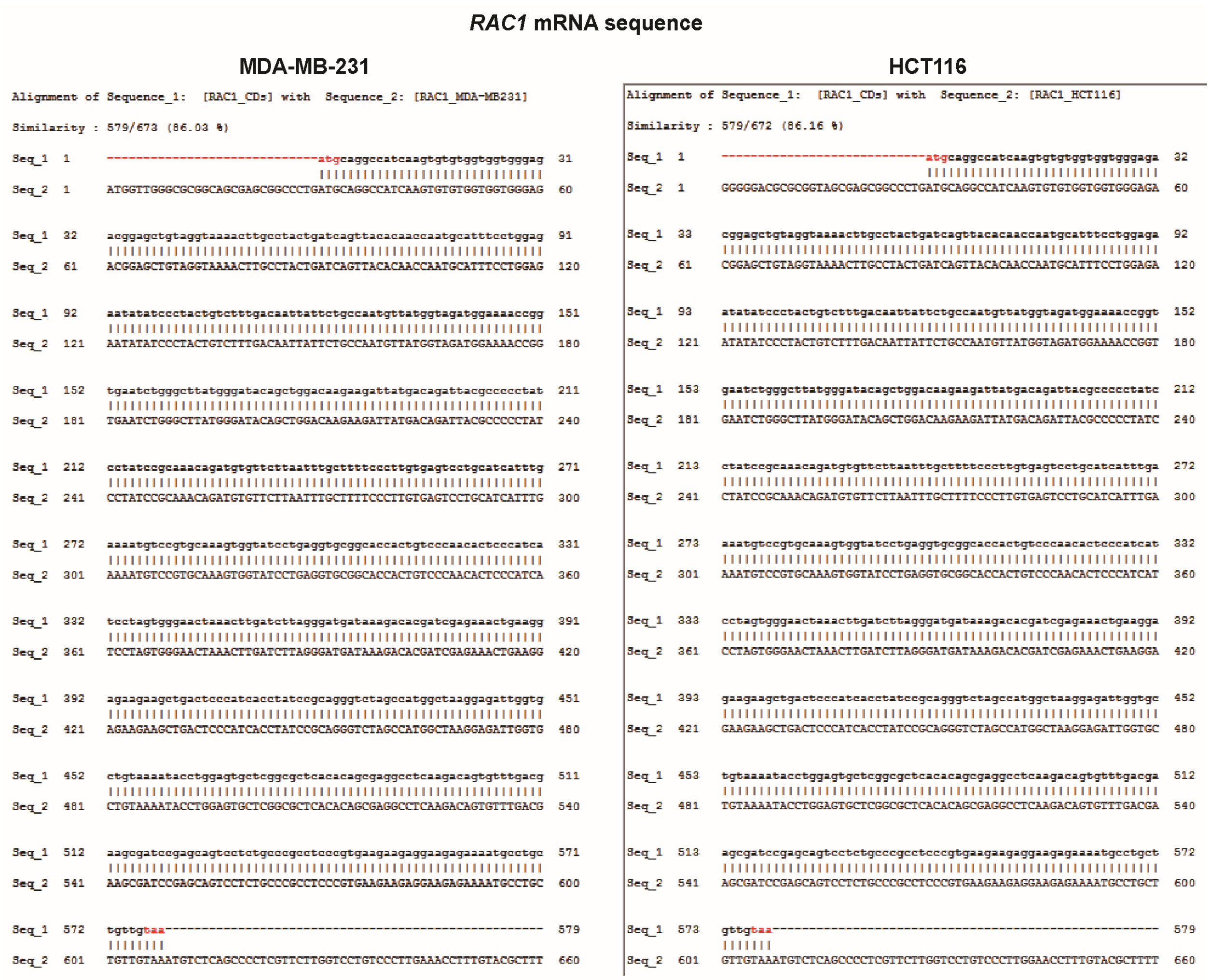

Figure S5. *RAC1* cDNA sequencing performed using total RNA derived from MDA-MB231 and HCT116 cells.

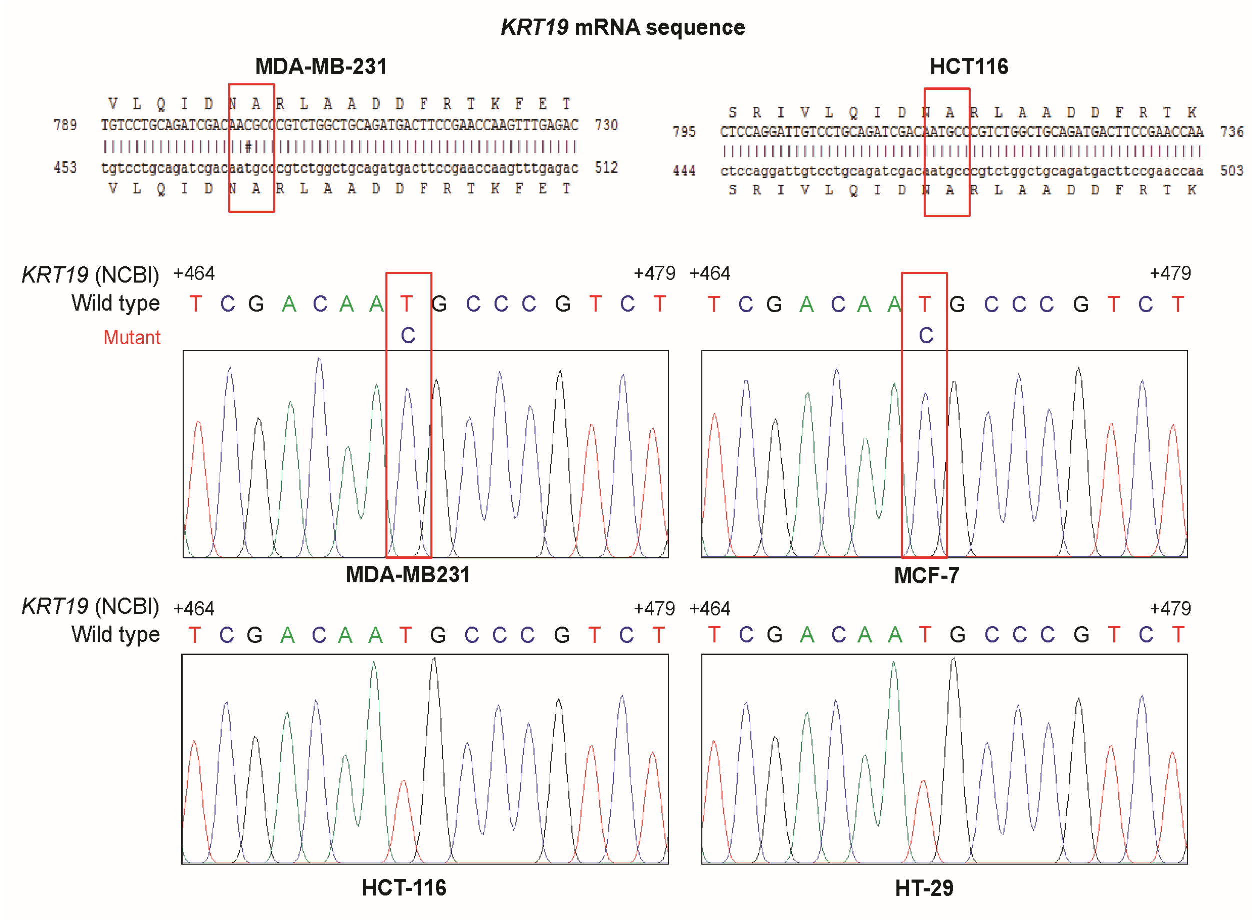

Figure S6. *KRT19* cDNA sequencing performed using total RNA derived from MDA-MB231, MCF7, HCT116, and HT29 cells. Chromatograms are shown for specific regions of sequences, and point mutations are indicated by red blocks.

© 2019 by the authors. Submitted for possible open access publication under the terms and conditions of the Creative Commons Attribution (CC BY) license (http://creativecommons.org/licenses/by/4.0/).
